# Physical activity and sedentary behavior patterns in adults with Crohn’s disease versus matched controls: accelerometer and self-report findings

**DOI:** 10.1007/s11332-026-01880-w

**Published:** 2026-08-03

**Authors:** Whitney N. Neal, Erica A. Schleicher, Thomas W. Buford, Daniel Chu, Gregory Pavela, Dori Pekmezi, Robert W. Motl

**Affiliations:** 1 Department of Health Behavior, University of Alabama at Birmingham, Birmingham, AL USA; 2 Department of Medicine, University of Alabama at Birmingham, Birmingham, AL USA; 3 Division of Hematology Oncology, University of Pittsburgh, Pittsburgh, PA USA; 4 Birmingham/Atlanta VA GRECC, Birmingham, AL USA; 5 Department of Surgery, University of Alabama at Birmingham, Birmingham, AL USA; 6 Department of Kinesiology & Nutrition, University of Illinois Chicago, Chicago, IL USA

**Keywords:** Physical activity, Sedentary behavior, Crohn’s disease, Inflammatory bowel disease

## Abstract

**Background:**

Physical activity is a promising complementary, non-invasive strategy for managing Crohn’s disease, but habitual physical activity remains poorly understood and even less is known about sedentary behavior in this population.

**Aims:**

To compare physical activity and sedentary behavior volume and patterns among adults with Crohn’s disease versus demographically matched controls.

**Methods:**

Participants (*N* = 79; 38 Crohn’s disease, 41 controls) completed the Godin Leisure-Time Exercise Questionnaire and wore an accelerometer for 7 days. Primary analysis included 32 matched pairs; sensitivity analyses used all participants and only those with ≥ 4 valid accelerometer wear days. MANOVAs assessed group differences in physical activity and sedentary behavior volume (self-reported moderate-to-vigorous physical activity, accelerometer-measured minutes/day in light physical activity, moderate-to-vigorous physical activity, sedentary time, steps) and pattern (accelerometer-measured moderate-to-vigorous physical activity and sedentary bout frequency and duration).

**Results:**

Primary multivariate analyses revealed no significant group effect for overall activity volume (Wilks’ Λ = .72, *p* = .09) or pattern (Wilks’ Λ = .71, *p* = .22), including when both were modeled simultaneously (Wilks’ Λ = .61, *p* = .42). However, secondary and sensitivity analyses indicated Crohn’s disease participants spent less total time sedentary (≈50 min/day) and engaged in fewer sedentary breaks (*p*’s < .05). Moderate-to-vigorous physical activity did not differ significantly between groups.

**Conclusions:**

Adults with Crohn’s disease were not uniformly less active than controls, but secondary and sensitivity analyses suggested possible differences in sedentary behavior profiles. These findings support personalized physical activity recommendations for Crohn’s disease and might support interventions emphasizing frequent movement and sitting-time interruption rather than solely increasing moderate-to-vigorous physical activity.

## Introduction

Crohn’s disease (CD) is a chronic inflammatory bowel disease (IBD) of the gastrointestinal tract that often leads to systemic and extraintestinal complications, including fatigue, joint pain, arthritis, osteoporosis, [1] and reduced physical fitness [2–5]. These disease-related factors can significantly impair health-related quality of life (HRQoL) and may influence daily movement patterns, including both physical activity (PA) and sedentary behavior (SB). PA is defined as any bodily movement produced by skeletal muscles that leads to an increase in energy expenditure [6], whereas SB refers to waking behaviors characterized by sitting, reclining, or lying with low energy expenditure (≤ 1.5 METS) [7]. Although closely related, PA and SB represent distinct dimensions of movement behavior with partially independent health implications across populations.

Emerging evidence suggests that PA may contribute to symptom management and HRQoL in adults with CD [8]; however, habitual PA levels, and notably SB, remain poorly characterized in this population. This gap is relevant given that SB has been independently linked to increased cardiovascular morbidity and mortality in the general population, even among individuals who meet PA guidelines [9–11]. Moreover, adults with CD appear to carry an elevated risk of cardiovascular disease that is not fully explained by traditional risk factors [12], highlighting the potential importance of movement behaviors as modifiable targets for disease management.

Accurate assessment of PA and SB is essential for understanding their relationship with disease activity and for informing clinical recommendations. Self-report measures are widely used in both research and clinical settings due to their practicality and low cost. However, these tools are susceptible to recall bias, misclassification of activity intensity, and imprecise quantification of activity duration and type [13]. These limitations are particularly relevant in CD, where symptom variability may influence both perceived and actual engagement in PA. Device-based measures such as accelerometry offer a more objective and granular assessment of daily movement, capturing low-intensity PA and SB time with greater precision than self-reported measures alone. The concurrent use of self-report and device-based measures provides a more comprehensive understanding of movement patterns—with self-report capturing contextual and perceived aspects of behavior and accelerometry quantifying intensity and distribution––in both healthy and clinical populations [14]. Despite the recognized value of accelerometry, few studies have employed this method in adults with CD [4, 15, 17 –], and even fewer have integrated both self-report and accelerometer-based measures within the same cohort.

Our recent scoping review suggested that adults with CD engage in lower levels of PA compared to the general population [8]. However, many of these studies included small control groups, lacked demographic matching, and primarily enrolled participants in clinical remission, limiting generalizability. Moreover, prior work has largely focused on total activity volume, often overlooking key components of movement behavior such as the temporal structure and fragmentation of activity, including prolonged sedentary bouts, breaks in sitting time, and postural transitions [18]. These components of activity patterns are increasingly recognized as important determinants of health [19] and may be particularly relevant in CD, where symptoms may shape not only how much activity is performed, but how it is accumulated across waking hours. Together, this evidence supports conceptualization of PA and SB volume and pattern as distinct domains warranting simultaneous examination.

To address these methodological limitations, we conducted a comprehensive comparison of PA and SB in adults with CD versus matched controls, utilizing both self-report and accelerometer measures to characterize activity volume and behavioral patterns. To clarify terminology used throughout the study, operational definitions of accelerometer-derived PA and SB volume and pattern are presented in Table 1. We hypothesized that adults with CD would engage in lower PA volume and pattern compared to their counterparts without CD [16]. We further hypothesized that the CD group would exhibit greater SB volume and more prolonged SB patterns, independent of differences in PA. By examining PA and SB as related but distinct domains of movement behavior, this study aims to generate clinically relevant evidence to guide disease-specific activity recommendations and inform tailored interventions that promote healthier movement behaviors and improve HRQoL in CD.

**Table 1:** Physical activity and sedentary behavior variables from accelerometers included in analyses

Domain	Metric	Definition
Volume	SB time	Daily time spent sitting, reclining, or lying down as determined by < 100 cpm (minutes, proportion^†^)
	LPA	Daily time spent in low-intensity activity as determined by 100–1951 cpm (minutes, proportion^†^)
	MVPA	Daily time spent in activity > 1951 cpm (minutes, proportion^†^)
	Step count	Cumulative number of steps taken over a day
Pattern	Sedentary bout	Unbroken period ≥ 30 min engaged in sedentary behavior (< 100 cpm)
	Sedentary bout length	Duration of each sedentary bout, calculated as total bout minutes/number of bouts (minutes)
	Sedentary break	A non-sedentary period (i.e., > 100 cpm) in between two sedentary bouts
	Total time in sedentary bouts	Sum of all time contained within sedentary bouts (minutes)
	MVPA bout	Unbroken period ≥ 8 min above threshold within any 10-min window engaged in MVPA (≥ 1952 cpm)
	MVPA bout length	Duration of each MVPA bout, calculated as total bout minutes/number of bouts (minutes)
	Total time in MVPA bouts	Sum of all time contained within MVPA bouts (minutes)

*SB* sedentary behavior, *cpm* counts per minute, *LPA* light physical activity, *MVPA* moderate-to-vigorous physical activity

^†^Proportion: time at intensity level/wear time

## Materials and methods

### Participants

We recruited individuals aged 18 – 65 years with CD and demographically matched controls who were willing to complete seven-day accelerometer monitoring and online questionnaire assessments. CD diagnosis was self-reported and confirmed through medical records where available (approximately 88% of participants had confirmed CD). Control group participants—defined as individuals without a clinically confirmed diagnosis of CD—were matched with CD participants on biological sex, educational attainment, and age (± 5 years). Educational attainment (completion of post-secondary education vs. all other qualifications including none) was selected as a proxy estimate of socioeconomic status based on previous research [20]. The study was approved by the University of Alabama at Birmingham Institutional Review Board for Human Use (IRB-300011077).

### Procedures

Recruitment for the study occurred January–December 2024 through multiple channels, including flyers, word-of-mouth, and face-to-face outreach at an outpatient Digestive Health Clinic located at a local academic medical center. Similar methods were employed to identify controls, as well as referrals from CD participants. Interested individuals contacted research staff via phone, email, or an online pre-screening survey. Once eligible, participants received an email link to the informed consent and study survey assessing demographic and clinical information (e.g., disease activity, disease duration, surgical history for the CD group only) and PA. They were also mailed an ActiGraph GT3X-BT monitor to wear for seven days, along with detailed instructions and a daily log to record their accelerometer wear time. After the monitoring period, participants returned the accelerometer using a provided self-addressed stamped envelope.

### Measures

#### Disease activity

Clinical disease activity was assessed in the CD group using the Harvey Bradshaw Index (HBI) [21], a validated self-report tool that measures general well-being, severity of abdominal pain, number of liquid stools, presence of abdominal mass, and presence of extraintestinal complications based on the previous day. Clinical remission is defined as an HBI score ≤ 4, while scores ≥ 5 indicate active disease. The HBI can be further categorized into remission (≤ 4), mild disease activity (5–7), moderate disease activity (8–16), and severe disease activity (> 16).

#### Self-reported physical activity

Self-reported PA data were collected using the Godin Leisure-Time Exercise Questionnaire (GLTEQ) [22]. The GLTEQ has been previously used to estimate the amount of strenuous, moderate, and light PA performed for periods of 15 min or more during a typical week among adults with CD [23–27]. The GLTEQ also calculates a Health Contribution Score (HCS) by multiplying the frequencies of strenuous and moderate activity by 9 and 5 metabolic equivalents, respectively, and summing weighted scores. These scores can range from 0 to 98, with higher scores indicating a greater volume of moderate-to-vigorous PA (MVPA). The GLTEQ HCS can further be converted into categories of insufficiently active (< 14 units), moderately active (14–23 units), or sufficiently active (≥ 24 units) [28, 29 ]. The GLTEQ HCS classification system has been validated against accelerometry in non-CD clinical populations [30, 31 ].

#### Accelerometer-measured physical activity and sedentary behavior

Accelerometry data were collected using ActiGraph GT3X-BT monitors, as these devices have been validated in the general population [32] and utilized in previous studies examining PA patterns in adults with CD [4, 15, 17 –]. The accelerometer was worn on the nondominant hip for seven waking days, excluding water-based activities, to capture daily ambulatory PA and SB. Wear time was validated using participant logs. A valid day required ≥ 10 h of wear without ≥ 30 min of continuous zero counts. Participants with at least one valid day were included in the primary analyses, as previous research suggests that single-day monitoring produces stable group estimates [33]. Sensitivity analyses restricted to participants with ≥ 4 valid days are also reported.

Accelerometry data were processed in ActiLife (software version 6.13.3) using a sampling frequency of 30 Hz and one-minute epochs with the low frequency extension (LFE) filter to enhance detection of low-intensity movements [34]. Counts were classified as sedentary (< 100 counts/minute), light PA (LPA; 100–1951 counts/minute), or MVPA (≥ 1952 counts/minute) [35]. MVPA bouts were defined as ≥ 8 consecutive minutes above threshold within any 10-min window, and sedentary bouts as ≥ 30 consecutive minutes with ≥ 80% of minutes below threshold and < 5 consecutive minutes above [36]. Steps were derived from the non-LFE data, whereas counts during the non-wear period—defined as ≥ 90 consecutive minutes with zero counts, permitting up to 2 min of nonzero counts if preceded and followed by ≥ 30 min of continuous zeros—were excluded from the analysis. Primary accelerometer-derived outcomes included: minutes/day and percentage of wear time spent in LPA, MVPA, and SB; daily steps; and the frequency and duration of MVPA and sedentary bouts (Table 1).

#### Statistical analyses

All analyses were conducted using IBM SPSS Statistics, Version 30. Statistical significance was set a priori at *p* < 0.05. Results are reported as frequencies and percentages (%) for categorical variables and mean ± standard deviation (SD) for continuous variables unless otherwise noted.

**Fig. 1:**
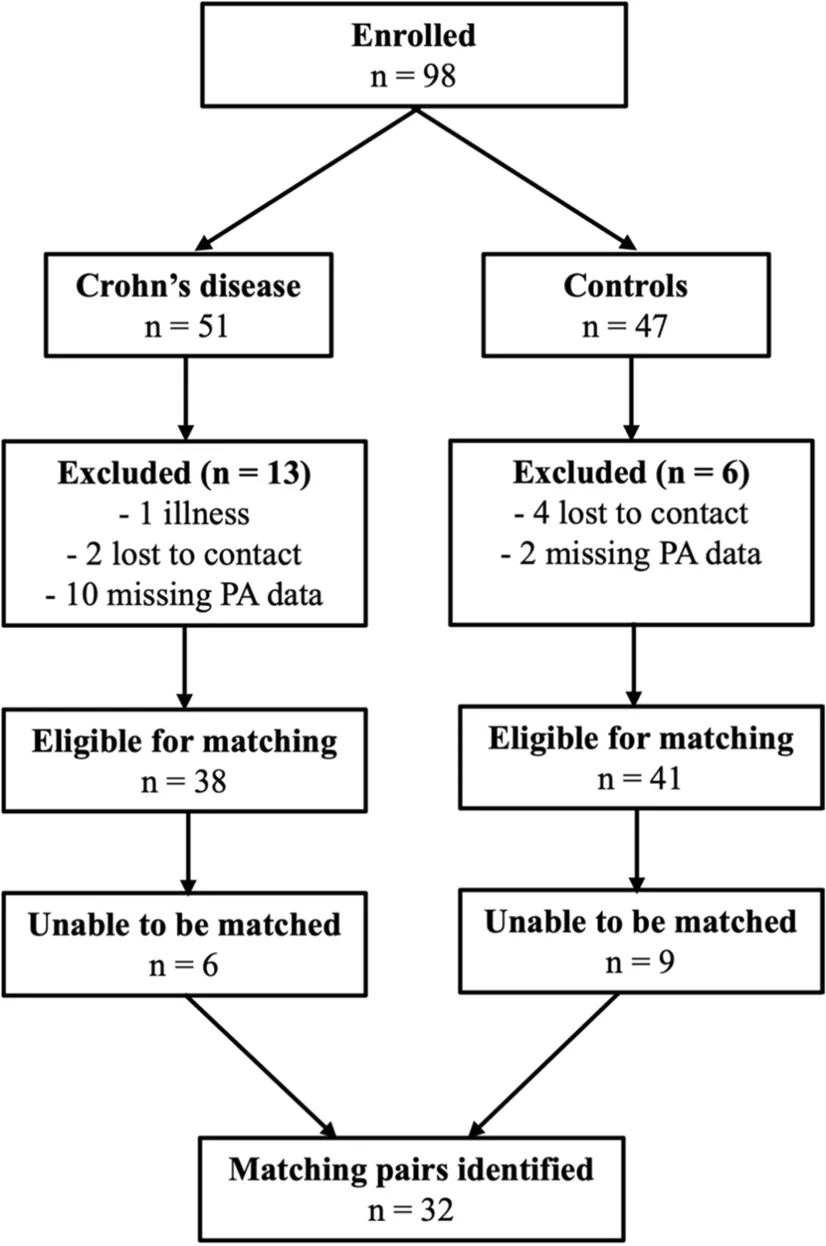
Flow of participant enrollment, exclusion, and matching. Of the 38 adults with Crohn’s disease and 41 controls eligible for matching, 6 individuals with Crohn’s disease could not be matched due to the unavailability of suitable control participants and were excluded from the primary analysis. The final analytic sample included 32 matched pairs (*N* = 64)

The initial sample for this study consisted of *N* = 92 participants (47 CD/ 45 controls) who were enrolled between January and December 2024 and completed data collection procedures. A total of 13 participants were ultimately excluded prior to the matching procedure due to missing valid accelerometer data, resulting in a pool of 79 participants (38 CD/ 41 controls) for the matching process. The remaining 38 CD participants were matched with a control participant based on sex, educational attainment (completion of post-secondary education vs. all other qualifications including none), and age (± 5 years). Six individuals with CD could not be matched due to the unavailability of a suitable pair from the control group and were excluded from the primary analysis. After accounting for unmatched participants, the final analytic sample consisted of 32 matched pairs (*N* = 64 participants). When examining differences between the analytic and excluded samples of CD and controls based on key demographic characteristics, participants in the unmatched group were more likely to be male (ϰ^2^ [1, *N* = 92] = 4.4, *p* = 0.04) and have not completed college (ϰ^2^ [1, *N* = 92] = 4.2, *p* = 0.04). However, no significant differences were found between the analytic and excluded samples of CD and controls based on age, race, income, or PA and SB outcomes, suggesting that missing data did not introduce substantial bias into our final sample. A CONSORT-style flow diagram detailing the enrollment and matching process is provided in Fig. 1.

Two-tailed paired samples t-tests and Wilcoxon signed-rank tests were performed to examine bivariate differences in self-reported MVPA and device-measured SB, LPA, and MVPA among individuals with CD and matched controls, standardized to total wear time. Effect sizes were quantified using Cohen’s *d* and interpreted as small, medium, and large based on values of 0.2, 0.5, and 0.8, respectively.

For the primary analysis, three one-way repeated measures multivariate analyses of variance (MANOVAs) were conducted to examine group differences across three domains: (1) activity volume, including self-reported MVPA, accelerometer-measured average minutes/day spent in LPA, MVPA, SB, and total steps; (2) activity patterns, reflected in the number and duration of MVPA and sedentary bouts; and (3) all PA and SB variables considered simultaneously in an omnibus repeated measures MANOVA. This analytical approach was selected over traditional MANOVA to account for the within-subject dependency among PA and SB volume and pattern, better model the intercorrelations among these variables, and improve sensitivity to group-level effects, particularly given the matched design and modest sample size. Wilks’ lambda (Λ) was used as the test statistic to assess the overall multivariate effect of the repeated measures factor. PA and SB variables were examined as related but distinct domains of movement behavior. Secondary analyses included follow-up Bonferroni-adjusted pairwise comparisons to examine domain-specific effects. Sensitivity analyses were conducted to examine the robustness of findings and included restricting analyses to participants with ≥ 4 valid days of accelerometer data. Although 52 participants met this criterion, only 23 complete matched pairs (*n* = 46 participants) were available for paired sensitivity analyses. Additional sensitivity analyses included the full unmatched sample (*N* = 79), and exploratory models excluding self-reported MVPA.

## Results

The characteristics of the CD and matched control participants who provided one or more valid days of accelerometry data are provided in Table 2 and demonstrate successful matching. The overall sample had a mean age of 40.0 years, was primarily composed of non-Hispanic, White females, and 65.6% had a college education. Among CD participants, the mean disease duration was 15.4 years, nearly two-thirds (65.6%) reported a history of bowel surgery, and fewer than half (40.6%) were in clinical remission according to the HBI. Of the 84.4% (*n* = 27) reporting medication use at the time of the study, 11.1% were receiving corticosteroids and 92.6% were on biologic therapy.

**Table 2:** Demographic and clinical characteristics by study group

Characteristic	Crohn’s Disease (*n* = 32)		Controls (*n* = 32)		*p* value
	n	%	n	%	
Sex (Female)	26	81.3	26	81.3	
Age (years; mean ± SD)	40.2 ± 11.9		39.7 ± 11.9		1.0
Disease duration (years; mean ± SD)	15.4 ± 10.9		N/A		
*Race*					.42^†^
White	20	62.5	23	71.9	
African American	10	31.3	5	15.6	
Asian	1	3.1	3	9.4	
Other (including multiracial)	1	3.1	1	3.1	
*Ethnicity*					1.0
Not Hispanic/Latino	31	96.9	31	96.9	
Hispanic/Latino	1	3.1	1	3.1	
*Education Level*					1.0
High school/ some college	11	34.4	11	34.4	
Technical/ college/ university degree	21	65.6	21	65.6	
*Annual Household Income*					.80
Less than $50,000	10	31.2	13	40.6	
More than $50,000	16	50.0	15	46.9	
Prefer not to answer	6	18.8	4	12.5	
*Current Medical Therapy (n* = *27)*^*‡*^					N/A
Corticosteroids	3	11.1	N/A	N/A	
Immunomodulators	1	3.7	N/A	N/A	
Biologics	25	92.6	N/A	N/A	
*Previous Bowel Surgery (n* = *21)*					N/A
Ostomy^§^	5	23.8			N/A
J-Pouch^§^	1	3.1			N/A
*Disease Activity (mean* ± *SD)*	6.9 ± 4.4		N/A		N/A
Remission (HBI ≤ 4)	13	40.6	N/A	N/A	
Mild (HBI 5–7)	5	15.6	N/A	N/A	
Moderate (HBI 8–16)	13	40.6	N/A	N/A	
Severe (HBI > 16)	1	3.1	N/A	N/A	

*HBI* Harvey Bradshaw index

^†^Between-group differences in race were computed using dichotomous categories (race: White vs. Black/other)

^‡^Respondents were allowed to report current use of any or all medications/treatments. Concurrent use of multiple medications is evident as total percent currently using medication/treatments is 107.4%

^§^Based on *n* = 21 participants who reported prior surgery

Normative data for Harvey Bradshaw Index: ≤ 4 = remission, 5–7 = mild, 8–16 = moderate, > 16 = severe disease activity

### Physical activity and sedentary behavior descriptives

**Fig. 2:**
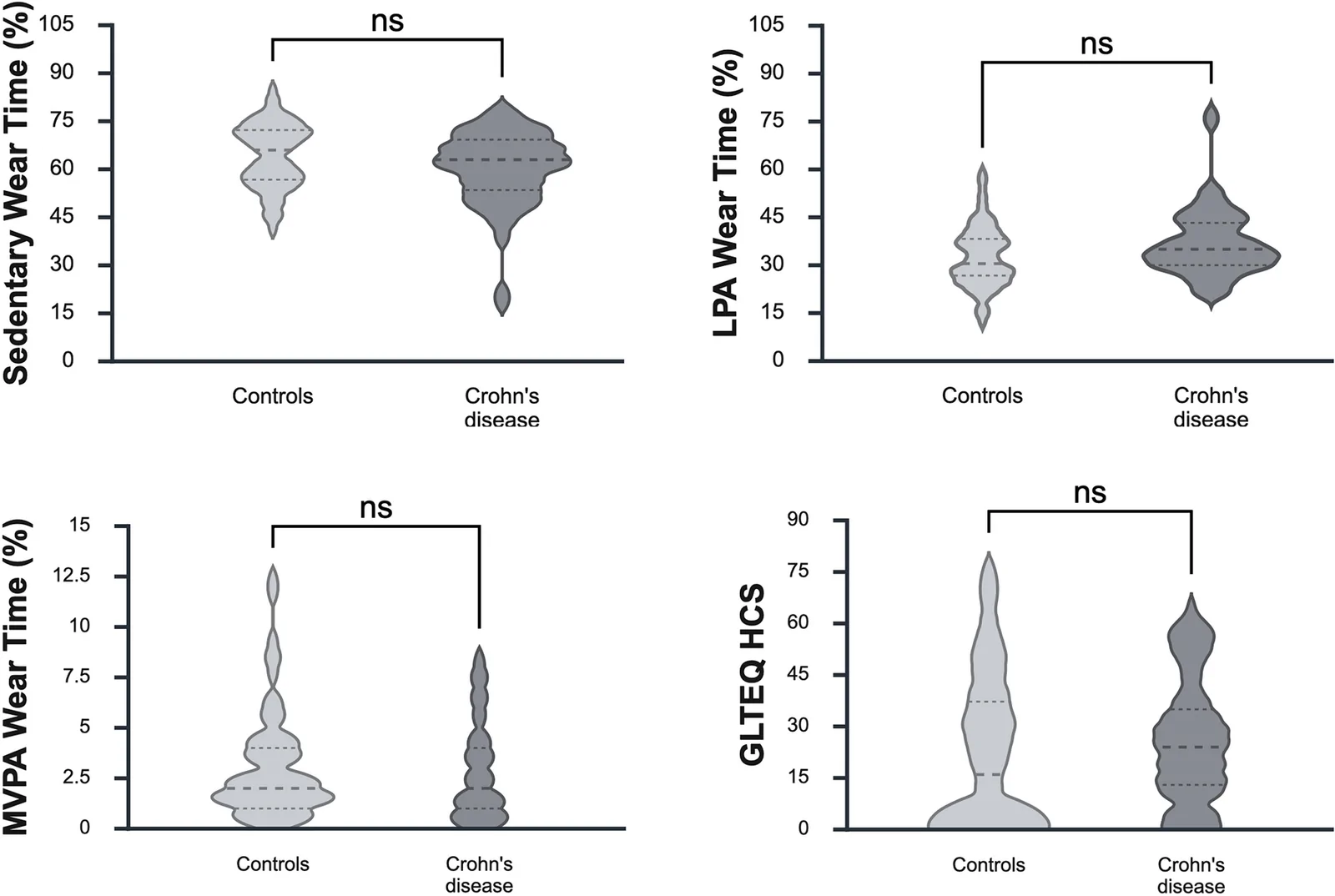
Activity volume outcomes expressed in percentage of wear time/week and self-reported moderate-to-vigorous physical activity for the Crohn's disease (*N* = 32) and matched control (*N* = 32) groups. Horizontal lines represent quartiles for weekly wear time percentages and Godin Leisure-Time Exercise Questionnaire Health Contribution Scores. *LPA* light physical activity, *MVPA* moderate-to-vigorous physical activity, *GLTEQ HCS* Godin Leisure-Time Exercise Questionnaire Health Contribution Score. Based on Wilcoxon signed-ranked tests. Created in BioRender. Neal, W.N. (2026) https://BioRender.com/g8fubdr

Descriptive statistics for PA and SB are presented in Table 3. Based on self-reported MVPA using the GLTEQ HCS, 75% of participants with CD were classified as “physically active” (GLTEQ HCS ≥ 24), compared to 53% of matched controls (χ^2^ [1, *N* = 64] = 3.33, *p* = 0.07). However, mean GLTEQ HCS scores did not differ significantly between groups (Fig. 2, *p* = 0.25), suggesting broadly comparable self-reported engagement in MVPA. For device-measured PA and SB, participants provided an average of 5.4 ± 1.8 days of valid accelerometer data, with 82.8% contributing ≥ 4 valid days. Mean daily wear time was comparable between groups (CD: 13.2 h/day; Controls: 13.5 h/day, *p* = 0.25). On average, CD participants spent 60% of their daily wear time in SB, 37% in LPA, and 3% in MVPA. Controls spent 64% in SB, 33% in LPA, and 3% in MVPA (Fig. 2).

**Table 3:** Descriptive statistics and one-way repeated measures multivariate analysis of variance results for activity volume and pattern in the Crohn’s disease and control groups (*N* = 64)

Measure	Crohn’s disease (*n = 32*)		Controls (*n* = 32)		*F*	η^2^_p_
	*M*	SD	*M*	SD		
*Volume*	Wilks’ Λ = 0.72, *F*(5, 27) = 2.15, *p* = .09					
SB time (min/day)	472.28	91.40	522.07	94.26	5.09*	.14
LPA (min/day)	294.34	94.04	264.94	89.52	1.60	.05
MVPA (min/day)	22.64	19.74	24.68	22.03	0.15	.01
Steps (per day)	6243.63	3230.47	5550.06	2539.99	0.89	.03
Self-reported MVPA^†^	25.19	18.49	21.31	22.88	0.48	.02
*Pattern*	Wilks’ Λ = .71, *F*(7, 25) = 1.48, *p* = .22					
Sedentary bouts	4.61	1.83	5.82	2.16	5.45*	.15
Sedentary bout length (min/day)	44.78	8.83	44.67	8.20	< 0.01	.00
Sedentary breaks	5.44	1.80	6.52	2.09	4.43*	.13
Total sedentary bouts (min/day)	221.53	97.86	276.53	116.05	3.90	.11
Active bouts	0.38	0.57	0.61	0.90	1.80	.06
Active bout length (min/day)	4.27	7.25	4.75	6.46	0.08	< .01
Total active bouts (min/day)	7.08	11.72	9.97	15.63	0.76	.02
Overall	Wilks’ Λ = .61, *F*(12, 20) = 1.09, *p* = .42					

*N* = 64 (*n* = 32 for each group). *SB*  sedentary behavior, *LPA *light physical activity, *MVPA* moderate-to-vigorous physical activity

^†^ As measured by the Godin Leisure-time exercise questionnaire health contribution score, possible range 0–98

^*^p < .05 based on Bonferroni-adjusted pairwise comparisons

Although percentage-based comparisons of time spent in SB, LPA, and MVPA were not statistically significant (Fig. 2), unadjusted pairwise comparisons indicated significant between-group differences in SB patterns. Compared to their matched counterparts, the CD group engaged in fewer bouts of SB (*M* = –1.21 bouts/day, *p* = 0.03) and had fewer breaks in SB time (*M* = –1.08 sedentary breaks, *p* = 0.04).

**Fig. 3:**
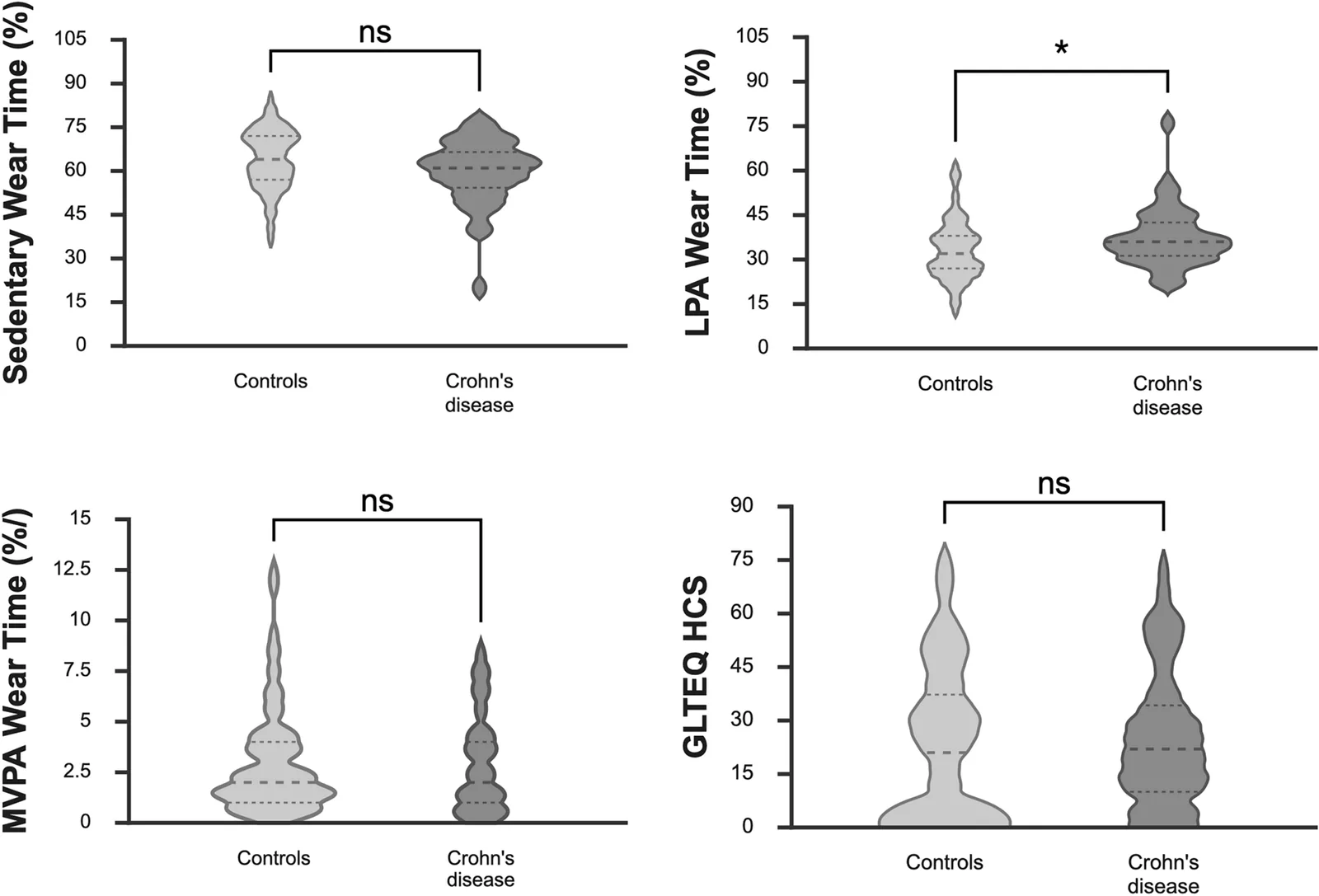
Activity volume outcomes expressed in percentage of wear time/week and self-reported moderate-to-vigorous physical activity for the full sample of Crohn's disease (*N* = 41) and control groups (*N* = 38). Horizontal lines represent quartiles for weekly wear time percentages and Godin Leisure-Time Exercise Questionnaire Health Contribution Scores. *LPA* light physical activity, *MVPA* moderate-to-vigorous physical activity, *GLTEQ HCS* Godin Leisure-Time Exercise Questionnaire Health Contribution Score. *p < .05 based on non-parametric Mann–Whitney U test. Created in BioRender. Neal, W.N. (2026) https://BioRender.com/g8fubdr

Results from a sensitivity analysis restricted to matched pairs with ≥ 4 days of valid accelerometer data (*n* = 23) further indicated statistically significant between-group differences in monitored time spent in SB as well as LPA. In this restricted sample, the CD group spent less wear time in SB (*M* = –7.4%, *p* = 0.04, *d* = 0.46) and more wear time in LPA (*M* =  + 7.7%, *p* = 0.02, *d* = 0.53) compared to their matched counterparts, suggesting lower SB volume was accompanied by greater engagement in light-intensity movement. Additional sensitivity analyses including the full sample of participants with complete self-report and at least one valid day of accelerometer data (total sample, *n* = 79, Table 4) were consistent with these results, as the between-group difference in monitored time spent in LPA was statistically significant based on equivalent bivariate tests (Fig. 3; *p*’s < 0.05).

**Table 4:** Demographic and clinical characteristics of the full sample by study group (*N* = 79)

Characteristic	Crohn’s Disease (*N* = 38)		Controls (*N* = 41)			*p*-value
	n	%	n	%		
Sex (Female)	30	78.9	30	73.2	.55	
Age (years; mean ± SD)	41.29 ± 11.97		39.29 ± 12.03		.59	
Disease duration (years; mean ± SD)	16.8 ± 11.4		N/A			
*Race*					.98^†^	
White	34	63.2	26	63.4		
African American	12	31.6	10	24.4		
Asian	1	2.6	3	7.3		
Other (including multiracial)	1	2.6	2	4.9		
*Ethnicity*					*.96*	
Not Hispanic/Latino	37	97.4	40	97.6		
Hispanic/Latino	1	2.6	1	2.4		
*Education Level*					.78	
High school/ some college	16	42.1	16	39.0		
Technical/ college/ university degree	22	57.9	25	61.0		
*Annual Household Income*					.59	
Less than $50,000	12	31.6	14	34.1		
More than $50,000	19	50.0	23	56.1		
Prefer not to answer	7	18.4	4	9.8		
*Current Medical Therapy (n* = *33)*^*‡*^						
Corticosteroids	3	9.1	NA	NA		
Immunomodulators	1	3.0	NA	NA		
Biologics	28	84.9	NA	NA		
*Previous Bowel Surgery (n* = *25)*			NA	NA		
Ostomy ^§^	6	24.0	NA	NA		
J-Pouch^§^	1	4.0	NA	NA		
*Disease Activity (mean* ± *SD)*^*¶*^	6.8 ± 4.3		NA			
Remission (HBI ≤ 4)	16	42.1	NA	NA		
Mild (HBI 5–7)	6	15.8	NA	NA		
Moderate (HBI 8–16)	15	39.5	NA	NA		
Severe (HBI > 16)	1	2.6	NA	NA		

*N = 79 (41 Crohn's disease, 38 controls). HBI* Bradshaw Index

^†^Between-group differences in race were computed using dichotomous categories (race: White vs. Black/other)

^‡^Respondents were allowed to report the use of any or all medications/treatments

^§^Based on *n* = 25 participants who reported prior surgery

^¶^Normative data for Harvey Bradshaw Index: ≤ 4 = remission, 5–7 = mild, 8–16 = moderate, > 16 = severe disease activity

### Multivariate analyses

One-way repeated measures MANOVA models revealed no significant main effects of study group for activity patterns (Wilks’ Λ = 0.71,* F*(7, 25) = 1.48, *p* = 0.22), activity volume (Wilks’ Λ = 0.72, *F*(5, 27) = 2.15, *p* = 0.09), or when all volume and pattern variables were modelled simultaneously (Wilks’ Λ = 0.61, *F*(12, 20) = 1.09, *p* = 0.42), Table 3. However, exploratory sensitivity analyses excluding self-reported MVPA (GLTEQ HCS) from the model indicated a significant effect of study group on activity volume (Wilks’ Λ = 0.72, *F*(4, 28) = 2.78, *p* < 0.05), suggesting that device-based volume metrics captured group differences not reflected in self-report (Table 5. Follow-up Bonferroni-adjusted bivariate comparisons from this exploratory sensitivity model indicated a significant group effect for SB, with CD participants spending approximately 49.7 fewer minutes/day sedentary than matched controls (95% CI –94.8 to –4.8; *p* = 0.04). No significant group differences were observed for daily steps, LPA, or MVPA, indicating that the multivariate effect was driven specifically by SB volume rather than higher-intensity accelerometer-derived activity.

**Table 5:** Descriptive statistics for activity volume and pattern in the full sample of Crohn’s disease and control groups

Measure	Crohn’s disease		Controls		*p*	
	*M*	SD	*M*	SD		*d*
Accelerometer wear time (hrs/d)	13.06	1.22	13.43	1.51	.24	.27
*Volume*						
*SB time (min/day)*	465.05	91.05	515.57	96.71	.02*	.54
LPA (min/day)	295.66	88.51	267.16	88.16	.16	.32
MVPA (min/day)	22.48	19.44	23.13	21.14	.89	.03
Step count	6223.67	3304.10	5642.03	2685.12	.39	.19
Self-reported MVPA^†^	24.32	18.53	24.29	23.49	.99	< .01
*Pattern*						
Sedentary bouts	4.76	8.28	5.67	2.23	.01**	.59
Sedentary bout length (min/day)	45.23	8.39	45.23	9.56	.99	< .01
Sedentary breaks	5.35	1.69	6.38	2.16	.02*	.54
Total time in sedentary bouts (min/day)	215.16	91.85	270.80	121.43	.03*	.51
Active bouts	.39	.55	.56	.85	.30	.24
Active bout length (min/day)	4.49	1.73	4.39	6.19	.82	.05
Total time in active bouts (min/day)	7.62	11.88	9.16	14.45	.61	.12

*N* = 79 (41 Crohn’s disease, 38 controls). *SB* sedentary behavior, *LPA* light physical activity, *MVPA* moderate-to-vigorous physical activity

^†^As measured by the Godin Leisure-Time Exercise Questionnaire Health Contribution Score, possible range 0–98

^*^p < .05; **p < .01 based on 2-tailed independent samples *t*-test

Sensitivity analyses restricted to participants with ≥ 4 valid days of accelerometer data (*n* = 46) revealed similar patterns, with additional significant main effects of study group for the activity patterns model (Wilks’ Λ = 0.36,* F*(7, 16) = 4.02, *p* = 0.01) along with the activity volume model excluding self-reported MVPA (Wilks’ Λ = 0.61,* F*(4, 19) = 3.03, *p* = 0.04). Follow-up Bonferroni-adjusted bivariate comparisons indicated significantly fewer minutes/day in SB (–61.8 (95% CI –111.75 to –11.78), *p* = 0.02), more minutes/day in LPA (+ 60.7 (95% CI 3.99 to 117.35), *p* = 0.04), and fewer sedentary bouts/day (–1.4 (95% CI –2.63 to –0.14), *p* = 0.03) in the CD group compared to controls. In the sensitivity analysis including the full sample (*n* = 79), significant main effects of study group were also observed for activity patterns (Wilks’ Λ = 0.82, *p* = 0.04). Follow-up Bonferroni-adjusted bivariate tests indicated significant differences in SB pattern outcomes (Table 6), further supporting the consistency of SB volume and pattern differences across analytic approaches.

**Table 6:** Multivariate analysis of variance results by volume of physical activity, pattern of physical activity, and volume and pattern combined in the full sample (*N* = 79)

Measure	*F*	*p*	η^2^_p_
*Volume* Wilks’ Λ = .92, *F*(5, 73) = 1.34, *p* = .26, η^2^_p_ = .08			
LPA (min/day)	2.05	.16	.03
MVPA (min/day)	0.02	.89	< .01
Sedentary time (min/day)	5.69	.02*	.07
Step count	0.74	.39	.01
Self-reported MVPA^†^	0.04	.84	< .01
*Pattern* Wilks’ Λ = .28, *F*(7, 71) = 2.24, *p* = .04*, η^2^_p_ = .18			
Sedentary bouts	6.81	.01*	.08
Sedentary bout length (min/day)	0.00	1.0	< .01
Sedentary breaks	5.66	.02*	.07
Total time in sedentary bouts (min/day)	5.21	.03*	.06
Active bouts	1.09	.30	.01
Active bout length (min/day)	0.05	.82	< .01
Total time in active bouts (min/day)	0.27	.61	< .01
*Overall* Wilks’ Λ = .80, *F*(12, 66) = 1.35, *p* = .21, η^2^_p_ = .20			

*N* = 79 (41 Crohn's disease, 38 controls). *LPA* light physical activity, *MVPA* moderate-to-vigorous physical activity

^†^As measured by the Godin Leisure-Time Exercise Questionnaire Health Contribution Score, possible range 0–98

^*^p < .05 based on Bonferroni-adjusted pairwise comparisons

## Discussion

This observational study sought to provide a nuanced understanding of PA and SB in adults with CD by comparing self-reported and accelerometer-measured activity patterns with those of demographically matched controls. Although primary multivariate analyses revealed no statistically significant differences in overall activity volume or patterns, follow-up secondary and sensitivity analyses consistently suggested differences in SB rather than in MVPA. Specifically, adults with CD spent less total SB time but also engaged in fewer sedentary-to-active transitions compared with controls. These findings underscore the importance of examining how SB is accumulated, rather than simply how much activity is performed, in chronic disease populations and of translating pattern insights into practical recommendations for clinical evaluation and treatment.

The absence of statistically significant differences in total activity volume or MVPA suggests many adults with CD may preserve higher intensity activity despite longstanding disease. These findings align with some prior studies reporting comparable self-reported MVPA levels between CD participants with minimal disease activity and their healthy counterparts [4, 15 ], whereas previous accelerometer-based work has reported lower monitored time in MVPA in CD participants (*Mdn* = 2.3%, range 0.3%–8.7%) compared to controls (*Mdn* = 3.8%, range 0.8%–8.3%; *p* < 0.01) [16]. In contrast, our matched design controlled for key demographic variables and included individuals across a broad range of disease activity, with almost half of CD participants in our analytic sample (43.7%; 14 of 32) meeting criteria for moderate-to-severe disease activity based on HBI scores, strengthening the internal validity and clinical relevance of these findings. Together, these methodological differences may help explain the inconsistent findings across studies and suggest that null MVPA findings should not be interpreted as an absence of meaningful behavioral differences, but rather as evidence that movement adaptations in CD may occur predominantly within other movement domains.

Consistent differences in SB patterns across univariate and sensitivity analyses further support this interpretation. Adults with CD spent less total time in SB and engaged in fewer sedentary bouts, yet also demonstrated fewer sedentary-to-active transitions compared to controls, suggesting altered regulation of SB rather than global inactivity. These findings somewhat contrast with the limited existing SB research in CD [4, 15, 17 –] and broader findings from other chronic disease populations [37, 38 ], which generally report greater SB time compared to healthy controls. SB is increasingly recognized as a behavioral construct with health implications that are partially independent of MVPA [7, 39, 40 ], and emerging evidence suggests that sedentary accumulation patterns, including prolonged bouts and infrequent interruptions, may be more strongly associated with adverse health outcomes than total SB time alone [19, 38 ]. Our findings suggest that SB patterns, specifically prolonged sitting punctuated by relatively infrequent sedentary-to-active transitions, may provide a useful characterization of daily movement behavior in adults with CD. Importantly, despite spending less monitored time in SB than controls (60% vs. 64%), SB time for both groups exceeded population-based estimates (55%, *p*’s < 0.001) [41]. Within CD care, these results suggest that evaluating SB volume and pattern may provide more actionable information for counseling and intervention design in CD care than MVPA metrics alone.

Our findings have practical implications for future intervention development. The observed trends in SB support approaches that promote exercise “snacks”—frequent, brief activity interruptions spread throughout the day [42]—rather than reliance on traditional structured, continuous exercise sessions. Such strategies may be more feasible during periods of symptom fluctuation and may better align with real-world behavioral constraints experienced by adults with CD. Future randomized controlled trials should compare intervention models targeting reductions in SB alongside traditional PA promotion, using device-derived volume and pattern metrics to clarify how different movement prescriptions influence symptom management and HRQoL.

The strengths of our study include the use of accelerometry with a validated self-reported measure and a demographically matched comparison group to examine movement behavior among adults with CD. We also enrolled participants across a range of disease activity, improving clinical applicability and increasing generalizability to typical CD populations. However, this study has several limitations that should be considered when interpreting results. The cross-sectional design inhibits the ability to establish a causal relationship, and the modest matched sample size and number of dependent variables included in the multivariate models limited statistical power to detect smaller group differences. While CD and control group participants were matched on multiple extraneous variables (biological sex, educational attainment, and age ± 5 years) to reduce confounding influence, we did not account for other potential factors (e.g., fatigue, anxiety, depression) that may influence activity behavior. Disease activity could not be included as a covariate in the primary repeated measures analyses because it was only assessed among participants with CD, and we did not stratify analyses by disease phenotype or symptom profile, which may have masked important subgroup differences. Future research should examine how activity patterns vary across CD phenotypes and whether certain disease characteristics are more strongly associated with beneficial activity patterns. Additionally, accelerometer data were not stratified by weekday versus weekend days, which may have limited detection of context-specific differences in PA and SB patterns. Although most CD diagnoses were confirmed through medical records, a small proportion (~ 12%) were based on self-report only, which may have introduced potential misclassification. Generalizability is further limited by 1) the predominantly female, well-educated CD sample recruited from a single academic medical center, 2) restriction to individuals under 65 years of age, 3) absence of a general health assessment for controls to rule out undetected or other health conditions that may have influenced activity patterns, and 4) the high prevalence of biologic therapy among CD participants (78% of the analytic sample), which may not represent the broader CD population and could influence activity behavior through symptom control.

In summary, this study contributes to a growing body of literature examining PA and SB in individuals with CD by integrating self-reported and accelerometer-based measures of both activity volume and accumulation patterns. While primary multivariate analyses did not demonstrate global group differences in activity behavior, secondary and sensitivity analyses suggested differences in SB patterns in the CD group, characterized by high SB time and few sedentary-to-active transitions. These findings underscore the value of device-based activity measurement in detecting clinically relevant behavioral features that may be missed by self-report alone and highlight the importance of evaluating SB as a distinct domain of movement behavior. As research and clinical care continue to move toward more personalized approaches to chronic disease management, understanding the nuances of PA and SB may be essential in developing realistic, evidence-based movement recommendations for adults with CD.

## Data Availability

The data underlying this article cannot be shared publicly due to privacy and ethical restrictions, specifically the potential for deductive disclosure of subjects given the unique characteristics of the study population. The de-identified data will be shared on reasonable request to the corresponding author.

## Appendix

See Table 4, 5, 6

Figure 3
